# Annotations

**Published:** 1890-08-02

**Authors:** 


### Annotations.

**Lady Novelists and Tall Men.**

A pathetic article appeared in last week's *Speaker* complaining of the decided and constantly-growing preference of lady novelists for tall heroes. According to the writer no lady who appeals to the public in fiction ever thinks of presenting a hero who is not tall and broad enough for the Life Guards. This same writer says he has reviewed about one hundred and ninety-two novels written by ladies, since last October. Happy reviewer! Each of those novels, of course, had a hero; one or more. Eighty-seven of the one hundred and ninety-two heroes stood six feet in their stocking soles, while many were considerably above that height. The average of the whole number was six feet three. It appears, according to this dejected writer, that the present average is about three quarters of an inch more than that of the preceding nine months, and that there has been for some time a steady tendency upwards. Is it really the fact that ladies generally set so much store by man nature when it is expressed in lineal yards? It must be so. No mere accident could have produced such wonderful unanimity in lady novelists. It is plain that if a woman could create her own ideal man, he would certainly be a Hercules in size, whatever he might be in respect of brains or character. But you are wrong ladies, you really are, in supposing that the biggest men are the best, and the most desirable for heroes and husbands. You know the old saying that the top storeys of tall houses are generally the emptiest. It is a common experience that very tall men have very weak heads; and though you may think that a weak-headed man is the easiest to manage, you are entirely mistaken. Such a man is often very difficult to manage; and what is much worse, he is not worth managing. The science of the matter is that men of the middle, or a very little over the middle height, are generally the healthiest, the best looking, the most active, the cleverest, and the sweetest-tempered of their kind. Of course, there are many exceptions. But it would be a better rule to choose a man a little under middle-sized than a great deal over for all-round work and wear and tear. You judge a great deal too much by the sight of the eyes, ladies! What would you say if every male novelist made his heroines five feet ten, and beautiful to boot? Some of you would have your heels made six inches high, and would be all day long gnashing your teeth with rage.

**Pasteurism and Politeness.**

Copies of two letters have been sent to us, one written by Mr. Victor Horsley, a Pasteurian, and the other by Dr. Dolan, an anti-Pasteurian. To those accustomed to reflect upon the quarrelling proclivities of Church fathers, metaphysicians, political economists, doctors, and all sorts of learned and "superior" persons, the quarrel is decidedly funny. A famous and witty preacher has declared that "in these times there is nobody infallible except young men." The letter of one of our two antagonists well illustrates the keen-witted preacher's aphorism. Mr. Victor Horsley cannot but be a young man since his bachelor's degree only dates from 1881. As a matter of fact he is a young man; and this is how he writes to a medical practitioner whose qualification dates from 1866 and who is a Fothergillian Gold Medallist, the winner of a £100 prize for an essay on "Life Assurance," and the holder of the Boylston Prize of the Harvard University. Says Mr. Horsley: "My attention has been drawn to a popular article on M. Pasteur and hydrophobia, written by you in the *Contemporary Review* for July, 1890. I took the trouble (*sic*) to correct in the *British Medical Journal* in 1866 numerous erroneous statements which you published on the same subject. . . . On February 13th, 1889, I read a paper on the subject before the Epidemiological Society. You were present. I therein proved that the death-rate of those bitten by unquestionably rabid dogs was 15 per cent., whereas the death rate among the same class of patients, when treated by M. Pasteur, was less than 1·6 per cent. . . . I am sending this letter to the medical journals for publication." The minatory tone adopted by the youthful Mr. Horsley towards his twenty years' senior is delightfully ludicrous. His "superiority," too, is exquisite. How Dr. Dolan dared to hold up his head, still less to appear in print after this terrible array of circumstances surpasses comprehension. To publish over again, and in a popular periodical, too, statements once corrected by Mr. Victor Horsley, and to dare to question conclusions that he had condescended to prove—that was an enormity which makes the plain man's hair stand as stiff and erect upon his head as a newly-cut wheat stubble. Dr. Dolan has his rejoinder, and whilst it is less minatory than that of Mr. Horsley, seeing that Dr. Dolan is not a young man, and therefore not infallible, it is adequately severe and highly dignified. Says Dr. Dolan: "M. Pasteur, in a popular magazine, the *New Review*, appealed to a public imperfectly educated in medical and scientific matters in support of his system. . . I think you rather under-estimate the intelligence of the public, to which you, the Pasteurians, did not hesitate to appeal at the Mansion House meeting. . . . You have ostentatiously labelled your own opinions as facts. . . . I prefer, even to your opinions, those of Professor Peter (Paris), Professor Billroth (Vienna), Professor Abieu (Portugal), Professors De Renzi and Amoroso (Naples), Professor Von Frisch (Vienna), Dr. B. W. Richardson (London), Vincent Richards (Calcutta), Dulles (Philadelphia), Herman Biggs (New York). . . . You can by manipulation of figures prove to your own satisfaction that M. Pasteur has reduced the mortality from 15 to 1·6 per cent. You might as well have said that he had reduced it from 65 or 93 to 1 per cent. . . . The problem now is, has M. Pasteur reduced the mortality from hydrophobia in the department of the Seine where he has been operating? If you care to defend this at the congress at Berlin, I shall reply to you in the negative." Thus stands the quarrel; and it very well represents the present state of scientific opinion on Pasteurism.

**Hume and Miracles.**

A Correspondent writes: "I wish you would refer me to the page in Hume's essay where you say he makes the statement you attribute to him. It is twenty years or more since I read the essay; therefore, I speak subject to correction. But I doubt whether he uses the words you put in his mouth. If I remember rightly, Hume says in effect, if not in words, that we have to trust to human testimony for the account of a miracle, and that it is more likely that this human testimony should be wrong than that a miracle should be true. This is very different from your statement." In the first place we did not put any words into Hume's mouth. We merely expressed the conviction that "At bottom the real position of Hume was that miracles could not take place, and that, therefore, they never had taken place." That is still our conviction; and we have arrived at it by personal discussion with Hume's disciples as well as by reading, and by a prolonged consideration of the frequent utterances of Hume's latest editor, Professor Huxley. The real words of Hume—we quote from memory—were: "It is not contrary to experience that testimony should be false, but it is contrary to experience that a miracle should be true." Exactly! It was contrary to David Hume's experience. But at the very moment when Hume wrote the sentence there were nearly 1,200 millions of other human beings alive, each of whom had a separate experience, quite different from his. Within historical times, tens of thousands of millions of human beings have lived, each with a separate experience. The fact of the matter is, that from the standpoint of inductive science, Hume's argument is not of the value of a feather's weight. It is a mere piece of *a priori* special pleading. Hume advanced it when induction was at a low ebb in the general mind, and more especially in the clerical mind. It would be just as scientific for one of Stanley's Africans to say to a European: "It is not contrary to experience that white men should tell lies, but it is contrary to experience that water should become solid, so that a man can walk on it without sinking." The present writer had a native African youth in his house for some months, a year or two ago. He repeatedly said, with deep amusement, that none of his fellow natives, when he should return to Africa, would believe him if he should tell them that he had often walked and skated on solid water, that is, upon ice. We cannot but repeat our conviction, that a skilful dialectician like Hume knew perfectly well the exceedingly flimsy character of his own argument. Being a philosopher, he could not bluntly say, "I don't believe in your pretended miracles, and there's an end of the matter." He was bound to invent an argument which should have at least the appearance of plausibility. That he could not invent a better one than he did, says little for the innate strength of the anti-revelationist's position.

